# Evaluating the role of a galanin enhancer genotype on a range of metabolic, depressive and addictive phenotypes

**DOI:** 10.1002/ajmg.b.32270

**Published:** 2014-09-16

**Authors:** Tom G Richardson, Camelia Minica, Jon Heron, Jeremy Tavare, Alasdair MacKenzie, Ian Day, Glyn Lewis, Matthew Hickman, Jacqueline M Vink, Joel Gelernter, Henry R Kranzler, Lindsay A Farrer, Marcus Munafò, David Wynick

**Affiliations:** 1 School of Social and Community Medicine, University of BristolBristol, UK; 2 Department of Biological Psychology/Netherlands Twin Register, VU UniversityAmsterdam, The Netherlands; 3 School of Biochemistry, University of BristolBristol, UK; 4 School of Medical Sciences, Institute of Medical Sciences, Foresterhill, University of AberdeenAberdeen, Scotland, UK; 5 Division of Psychiatry, University College LondonLondon, UK; 6 Department of Psychiatry, Genetics, and Neurobiology, and VA CT Healthcare Center, Yale University School of MedicineWest Haven, Connecticut; 7 MRC Integrative Epidemiology Unit, UK Centre for Tobacco and Alcohol Studies, and School of Experimental Psychology, University of BristolBristol, UK; 8 Schools of Physiology and Pharmacology and Clinical Sciences, University of BristolBristol, UK

**Keywords:** galanin, cannabis, alcohol, metabolism, depression, ALSPAC

## Abstract

There is a large body of pre-clinical and some clinical data to link the neuropeptide galanin to a range of physiological and pathological functions that include metabolism, depression, and addiction. An enhancer region upstream of the human *GAL* transcriptional start site has previously been characterised. In-vitro transfection studies in rat hypothalamic neurons demonstrated that the CA allele was 40% less active than the GG allele in driving galanin expression. Our hypothesis was to investigate the effect of this galanin enhancer genotype on a range of variables that relate to the known functions of the galaninergic system in the Avon Longitudinal Study of Parents and Children (ALSPAC) cohort of young adults (N = 169–6,078). Initial findings showed a positive relationship of cannabis usage (OR = 2.070, *P* = 0.007, N = 406 (individuals who had used cannabis at least once within the last 12 months, total sample size 2731) with the GG haplotype, consistent with the previous published data linking galanin with an increased release of dopamine. As our sample size was relatively small we replicated the analysis in a larger cohort of 2,224 African Americans and 1,840 European Americans, but no discernible trend across genotypes was observed for the relationship with cannabis usage. Further, we found no association of the galanin enhancer genotype with any of the other pathophysiological parameters measured. These findings emphasise that preclinical data does not always predict clinical outcomes in cohort studies, noting that association studies are subject to multiple confounders.

## INTRODUCTION

The neuropeptide galanin (Tatemoto et al., 1983) has a discreet pattern of expression in a number of areas in the central and peripheral nervous system that include the hypothalamus, hippocampus, substantia nigra, ventral tegmental area, nucleus accumbens (NAc), locus coeruleus, spinal cord, and the dorsal root ganglia (Melander et al., 1985; Melander et al., 1986a; Melander et al., 1986b; Hokfelt et al., 1987; Villar et al., 1989; Wiesenfeld et al., 1992). There is a wealth of pharmacological and genetic pre-clinical data to link the neuropeptide and its three G-protein coupled receptors (Gal_1_, Gal_2_, and Gal_3_) to a range of physiological and pathological functions. These include metabolism, feeding and endocrinology, cognition, epilepsy, chronic anxiety and depression, addiction, neuroprotection, neuronal regeneration, and pain (see recent reviews (Lang et al., 2007; Ogren et al., 2010; Picciotto et al., 2010)). Some, but by no means all, of these rodent findings are paralleled by human studies demonstrating associations between single nucleotide polymorphisms (SNPs) in the *GAL* gene and/or one of its three receptors, and depression or anxiety disorders (Unschuld et al., 2008; Wray et al., 2010; Juhasz et al., 2014) and addictive behaviours that include smoking (Gold et al., 2012), alcohol (Belfer et al., 2006), and heroin (Levran et al., 2008). To date, there are no studies that link the galaninergic system to dietary fat intake or weight regulation in humans, though a single study has shown a relationship with elevated triglyceride levels in familial combined hyperlipidaemia (Plaisier et al., 2009).

Davidson et al. previously used comparative genomics to identify and then characterise a highly conserved region 42 kb upstream of the human *GAL* transcriptional start site that was termed GAL5.1 (Davidson et al., 2011). The GAL5.1 enhancer (i.e., a region of DNA which can be bound with proteins to activate transcription of a gene) contains two polymorphisms; rs2513280 (C/G) and rs2513281 (A/G), that occur in two allelic combinations (GG or CA) where the dominant GG allele occurred in 70–83% of the human population. In-vitro transfection studies in rat hypothalamic neurons demonstrated that the CA allele was 40% less active than the GG allele in driving galanin expression (Davidson et al., 2011). Both SNPs were found to be in linkage disequilibrium (LD) with another SNP (rs2156464) previously associated with major depressive disorder. More recently, the GG haplotype, which predicts greater galanin expression, has been shown to be associated with problem drinking in women (Nikolova et al., 2013).

Here we have used the Avon Longitudinal Study of Parents and Children (ALSPAC) cohort to study whether the galanin enhancer haplotype is linked to a range of variables that relate to the known pre-clinical functions of the galaninergic system. These include body mass index, blood pressure, cognition, anxiety, and depression scores and usage of alcohol, cannabis, and smoking. Our initial findings demonstrated a relationship of cannabis usage with the GG haplotype, which predicts greater galanin expression (Davidson et al., 2011). These findings however were not replicated in either the Young Netherlands Twin Register (YNTR) or the Yale-UPenn study, a large cohort of African (AAs) and European Americans (EAs). Further, and despite the significant rodent pre-clinical data sets, we found no association of the galanin enhancer haplotype with the other pathophysiological parameters measured.

## MATERIALS AND METHODS

### Cohort Descriptions

The ALSPAC study uses a population-based cohort to investigate genetic and environmental factors that affect the health and development of children. The study methods have been described in detail elsewhere (Boyd et al., 2013) (http://www.bristol.ac.uk/alspac). Briefly, 14,541 pregnant women residents in the former region of Avon, UK, with an expected delivery date between 1st April 1991 and 31st December 1992, were eligible to take part in ALSPAC. There were 14,062 live born children, 13,988 of whom were alive at 1 year. Ethical approval was obtained from the ALSPAC Law and Ethics Committee and the Southmead, Frenchay, UBHT and Weston Research Ethics Committees. Written informed consent was obtained from parents for all measurements made.

YNTR is a population based study including more than 70,000 Dutch twins and their siblings, born after 1985 (van Beijsterveldt et al., 2013). Briefly, the YNTR participants are registered at birth and phenotyped longitudinally in surveys focused on behaviour problems, health, and lifestyle. Up to the age of 14, the surveys are completed by parents and teachers. From age 14 onward, the data are collected by self-report, based on prior parental written consent. The study protocol was approved by the Medical ethical committee of the VU Medical Centre, Amsterdam, The Netherlands (IRB00002991). For the present replication study, a sample of 362 individuals (mean age = 17.96; range = 14–27.5 years; 51.4% males) was available. For these subjects both genotype data were available as well as data on cannabis frequency. If more than one time point was available, data collected in the most recent survey were included in this analysis.

The Yale-UPenn study included 1,840 EA and 2,244 AA subjects who had both genotype data and measures of cannabis use. All subjects were recruited for studies of the genetics of drug (opioid or cocaine) or alcohol dependence (Gelernter et al., 2013). The sample consisted of small nuclear families (SNFs) originally collected for linkage studies (primarily full sibs, half sibs, and parents, generally no more than one parent per family) and unrelated individuals who were recruited in the Eastern US (CT, PA, MA, and SC). Subjects gave written informed consent as approved by the institutional review board at each site, and certificates of confidentiality were obtained from NIDA and NIAAA.

### Data Collection

We initially identified four broad areas that galanin has been linked to in previous epidemiological studies; metabolism, cognition, depression, and addiction (see recent reviews (Lang et al., 2007; Ogren et al., 2010; Picciotto et al., 2010)). We then selected traits from the phenotypically-rich ALSPAC cohort which best represented these epidemiological areas of interest (see below and supporting information).

### Metabolism

Height was measured to the nearest 0.1 cm using a Leicester height measure (Holtain Crosswell, Dyfed,UK) and weight was measured to the nearest 0.1 kg using Tanita electronic scales. Waist circumference was measured to the nearest 1 mm at the mid-point between the lower ribs and the pelvic bone with a flexible tape. Whole body dual-emission X-ray absorptiometry (DXA) determined fat and bone mass was assessed at the clinic using a Lunar Prodigy scanner (GE Lunar, Madison, WI) with paediatric scanning software.

Blood Pressure was measured with a Dinamap 9301 vital monitor completed by trained staff using the appropriate cuff size. Two readings of both systolic and diastolic blood pressure were taken when the study participants were at rest and the mean of each were used as a measurement in our analysis. Fasting blood samples were taken from participants who attended the age14 clinic (median age: 15 years and 5 months, IQR: 15 years and 3 months–15 years and 7 months) after they had been asked to fast for at least six hours and then immediately spun and frozen at −80°C. Measurements were then assayed three to nine months after the samples were taken. Plasma lipid concentrations (including total cholesterol and triglycerides) were measured by modification of the standard lipid research clinics protocol with enzymatic reagents for lipid determination (Myers et al., 2000).

### Cognition

Measurements for participants reading level were taken at the age 7-clinic (median age: 7 years and 5 months, IQR: 7 years and 5 months–7 years and 6 months) using the Wechsler Objective Reading Dimensions (WORD) test (Rust et al., 1993). The following cognitive function measures were recorded at the age 8 clinic (median age: 8 years and 7 months, IQR: 8 years and 6 months–8 years and 8 months). IQ was measured using the Weschler Intelligence Scale for Children (Weschler et al., 1992) (3rd UK edition (WISC–III). A shortened version of the scale was applied by trained psychologists who used alternate items for all subtests (with the exception of the coding subtest) in the scale. Short-term memory was also measured at the age 8 clinic using an adaption of the Nonword Repetition Test (Gathercole et al., 1994). Participants were asked to repeat 12 nonsense words comprised of three, four or five syllables after hearing them on an audio cassette. The outcome variable was recorded as the number of words repeated correctly. Speech and language were tested using items from the oral expression and language comprehension subsets of the Wechsler Objective Language Dimensions (Rust, 1996) (WOLD). At the age 10 clinic (median age: 10 years and 7 months, IQR: 10 years and 5 months–10 and years and 9 months) working memory was assessed using the Counting Span working memory task (Case et al., 1982) which involved counting and recalling numbers of dots on a screen. Two scores were recorded as separate variables, the span score which represents the number of correctly recalled sets of dots (maximum score of 5 in increments of 0.5) and a global score based on the number of screens correctly answered (maximum score of 42).

### Depression

Four thousand seven hundred thirty eight individuals who attended the age 17 clinic (median age: 17 years and 9 months, IQR: 17 years and 7 months–17 years and 11 months) completed a computerised self-reported questionnaire for the revised version of the Clinical Interview Schedule (CIS-R). CIS-R has been designed to identify the nature and severity of any neurotic symptoms or presence of neurosis experienced over the previous seven days (Lewis et al., 1992). A variable was then generated using the five depression symptom scores to get a continuous depression score. We dichotomised this variable to obtain a binary trait, where scores greater than 12 were classed as a clinically significant levels of distress. Furthermore, the ICD-10 diagnosis was also derived from the CIS-R data which allowed us to generate a categorical variable for analysis (Organization, 1993).

Participants were also invited to a PLIKS (psychosis like symptoms) semi-structured interview (Horwood et al., 2008) (PLIKSi) at the age 11 clinic (median age: 11 years and 9 months, IQR: 11 years and 7 months–11 years and 10 months). The interview consisted of 12 core questions concerning the occurrence of hallucinations, delusions, and experiences of thought interference over the past six months. For more detail, seven of the questions were derived with slight modification from DISC-IV (Shaffer et al., 2000) and the other five from section 17 of the Schedules for Clinical Assessment in Neuropsychiatry (SCAN) version 2.0 (Organization, 1994). The interviewer categorised each PLIKs as either “not present”, “suspected” or “definitely present”, which were coded as 0, 1 or 2 respectively and combined to make an overall score.

### Addiction

At both the age 14 clinic (median age: 15 years and 5 months, IQR: 15 years and 3 months–15 years and 7 months) and the age 17 clinic (median age: 17 years and 9 months, IQR: 17 years and 7 months–17 years and 11 months) data on self-reported alcohol intake, cannabis use and frequency of tobacco smoking in the past month was collected. A subset of participants (N = 2,933) at the age 17 clinic had two cotinine level measurements (ng/ml) taken which were assessed using immunoassay from blood plasma and the mean was used as a measurement in our analysis. As we were interested in quantifying addiction we removed all non-smokers from subsequent analyses (N = 1,428). A dichotomised phenotype was also calculated for this data using a cut-off of 50 ng/ml in order to distinguish daily smokers from non-daily smokers, based on data from a Finnish sample that used similar methods for cotinine assay (Vartiainen et al., 2002).

Also at the age 17 clinic, participants were asked to complete a six item cannabis abuse screen test (CAST) (Legleye et al., 2012) which entailed questions about cannabis use in the past year. This outcome was dichotomised for our analysis to distinguish between heavy cannabis users (i.e., a CAST score of one or more) and infrequent cannabis users. The problem gambling severity index (PGSI, derived from the longer Canadian Problem Gambling Inventory (Miller et al., 2013)) was administered to participants who had reportedly engaged in any of 16 types of gambling in the past year. A PGSI score of 1 or more was used to indicate a participant who had shown signs of problem gambling in that time.

Yale-UPenn: Subjects were administered the Semi-Structured Assessment for Drug Dependence and Alcoholism (SSADDA) (Pierucci-Lagha et al., 2005) to determine the frequency of cannabis use and time spent drinking heavily (detailed phenotype description below) as well as other major psychiatric traits. Subjects with bipolar affective disorder or schizophrenia were excluded, except as relatives. All subjects signed written informed consent as approved by IRBs at each clinical site; and certificates of confidentiality were obtained from NIAAA and NIDA.

### ALSPAC Genotyping

Davidson et al., [2011] previously analysed the GAL5.1 haplotype region (rs2513280 (C/G) and rs2513281 (A/G)). However, these two SNPs are in perfect LD in European populations (R^2^ = 1.00 and D’ = 1.00 in CEU according to HapMap) which makes rs2513280 a perfect proxy for the GAL5.1 haplotype. All genotyping was performed by KBiosciences (Herts, UK) using their own system of fluorescence based competitive allele-specific PCR (KASPar). Details of assay design are available from the KBiosciences website (http://www.kbioscience.co.uk). 8,365 offspring were successfully genotyped for the rs2513280 (*GAL*) variant. Study participants with a reported non-white ethnic background (N = 21) were excluded from analyses. A further 48 offspring were removed as they were either the second born child or born in a multiple pregnancy. This left a sample size of 8,296 which was in the Hardy-Weinberg Equilibrium (χ^2^ = 0.26, *P* = 0.610).

### Statistical Analysis

ALSPAC: Pearson's χ^2^-test was used to examine if the genotype distributions were consistent with Hardy–Weinberg equilibrium (HWE). Genotypes were coded as 0, 1 or 2 based on the number of G alleles, as Davidson et al., [2011] reported the GG haplotype to be associated with higher activity of galanin. Linear regression was used to investigate per-allele associations of rs2513280 with continuous phenotypes. Multinomial logistic regression was applied to calculate odds ratios (OR) for categorical traits using the addictive model of inheritance. Phenotypes that were not normally distributed were log transformed on the log 10 scale to approximate univariate normality before conducting any analyses. Multiple testing was corrected for using the Bonferroni correction (Bonferroni, 1936). Any possible interaction between genotype and sex was assessed using the likelihood ratio test to compare two regression models, one which was simply adjusted for sex and another which also included an interaction term for genotype*sex. If this analysis provided evidence of an interaction we analysed males and females separately to verify whether any association appeared when stratifying by sex. This was of particular interest for any relationship identified between rs2513280 and alcohol as there has been a previously reported gender-specific association with problem drinking at this locus (Nikolova et al., 2013). Stata 12.0 software (StataCorp, College Station, TX) was used for all statistical analyses.

YNTR: The analysis was performed in Plink (Purcell et al., 2007) by using a logistic regression model. As the sample included sibships of sizes 1–4, consisting of monozygotic and dizygotic twins and additional siblings, a sandwich correction (Williams, 2000) was used to take data clustering into account. Sex and age at the last survey and three principal components were included as covariates.

Yale-UPenn: Two phenotypes were tested in the US cohort. The first was a binary variable indicating whether, at the time of subject's lives during which they were using cannabis most heavily, they used the drug 13 or more times per month. Association was tested with rs2513280 using logistic regression models adjusted for age, sex, and three principal components of ancestry. The SNP was tested separately in EAs and AAs. Generalized estimating equations were used to correct the standard error for the fact that the sample contained related individuals.

## RESULTS

### Metabolism

Body mass index and waist circumference were analysed as continuous traits at six different time points (Tables1 and 2), and there was no evidence that rs2513280 was associated with either of these phenotypes. Systolic and diastolic blood pressure were analysed as continuous traits across four time points but there was also little evidence of an association or discernible trend across genotypes (Table3). Dual-energy X-ray absorptiometry (DXA)-assessed fat and bone mass was analysed at four time points and although the results for fat mass were negative there was an addictive trend across genotypes at ages 9 and 13 for bone mass (*P* = 0.014 and 0.023 respectively, Table4). However, these results were not strong enough to survive the correction for multiple comparisons at a 5% level. Fasting triglycerides were analysed as a continuous traits but the result were not associated with rs2513280 (*P* = 0.207). We then used the 90^th^ percentile to classify cases and controls for hypertriglyceridemia (the same cut-off as used by Plaisier et al., 2009). There was an increased odds per allele towards the G allele, suggesting that higher galanin expression contributes to increased risk of hypertriglyceridemia, although the sample was too small to identify evidence of an association (OR: 1.15 (95% CI 0.90–1.47) *P* = 0.267, N = 2863).

**Table I:** Association of rs2513280 (*GAL)* Genotypes with BMI (kg/m^2^) in the ALSPAC Cohort

Mean age (years)	N	C/C	C/G	G/G	*P*_add_	*P*_dom_	*P*_rec_
7.6	6,075	16.178 (1.922)	16.199 (2.082)	16.239 (2.023)	0.416	0.811	0.403
9.9	5,819	17.722 (2.755)	17.655 (2.893)	17.687 (2.810)	0.729	0.817	0.640
10.7	5,622	18.153 (2.852)	18.212 (3.104)	18.201 (3.060)	0.977	0.942	0.955
11.8	5,421	18.777 (3.100)	19.109 (3.462)	19.026 (3.337)	0.861	0.412	0.643
13.9	4,746	19.880 (3.137)	20.388 (3.477)	20.327 (3.442)	0.801	0.162	0.856
15.5	4,176	21.182 (3.490)	21.363 (3.530)	21.413 (3.500)	0.468	0.515	0.548

Genotype data are presented as mean (standard deviation).

*P*_add_, *P*-value for addictive effect (C/C = 0, C/G = 1, G/G = 2); *P*_dom_, *P*-value for dominant effect (C/C = 0, C/G= 1, G/G = 1); *P*_rec_, *P*-value for recessive effect (C/C = 0, C/G = 0, G/G = 1).

**Table II:** Association of rs2513280 (*GAL)* Genotypes With Waist Circumference (cm) in the ALSPAC Cohort

Mean age (years)	N	C/C	C/G	G/G	*P*_add_	*P*_dom_	*P*_rec_
7.6	6,078	56.357 (4.731)	56.293 (5.219)	56.467 (5.161)	0.266	0.948	0.219
9.9	5,867	62.830 (7.638)	62.787 (7.855)	62.894 (7.671)	0.600	0.958	0.566
10.7	5,659	65.269 (7.890)	65.476 (8.612)	65.523 (8.662)	0.799	0.823	0.832
11.8	5,423	67.487 (8.181)	68.401 (9.471)	68.204 (9.373)	0.836	0.429	0.625
13.9	4,741	70.872 (8.528)	72.114 (8.861)	72.050 (9.310)	0.707	0.170	0.973
15.5	3,454	75.574 (9.183)	76.533 (8.907)	76.645 (8.823)	0.383	0.275	0.531

Genotype data are presented as mean (standard deviation).

*P*_add_, *P*-value for addictive effect (C/C = 0, C/G = 1, G/G = 2); *P*_dom_, *P*-value for dominant effect (C/C = 0, C/G= 1, G/G = 1); *P*_rec_, *P*-value for recessive effect (C/C = 0, C/G = 0, G/G = 1).

**Table III:** Association of rs2513280 (*GAL)* Genotypes With Blood Pressure (mmHg) in the ALSPAC Cohort

Mean age (years)	N	Type	C/C	C/G	G/G	*P*_add_	*P*_dom_	*P*_rec_
7.6	6,013	Systolic	98.578 (8.550)	98.644 (9.058)	98.932 (9.259)	0.275	0.731	0.265
		Diastolic	55.799 (5.592)	56.409 (6.590)	56.301 (6.665)	0.986	0.358	0.749
9.9	5,806	Systolic	102.821 (8.875)	102.710 (9.280)	102.710 (9.280)	0.897	0.897	0.918
		Diastolic	57.959 (6.336)	57.431 (6.357)	57.359 (6.388)	0.393	0.298	0.537
11.8	5,378	Systolic	104.706 (10.054)	105.494 (9.858)	105.496 (9.810)	0.660	0.386	0.832
		Diastolic	58.399 (6.155)	58.899 (6.509)	58.659 (6.585)	0.452	0.594	0.309
15.5	4,087	Systolic	122.208 (10.398)	122.660 (10.928)	123.098 (10.890)	0.187	0.493	0.207
		Diastolic	67.609 (8.107)	67.512 (8.946)	67.412 (8.696)	0.709	0.850	0.720

Genotype data are presented as mean (standard deviation).

*P*_add_, *P*-value for addictive effect (C/C = 0, C/G = 1, G/G = 2); *P*_dom_, *P*-value for dominant effect (C/C = 0, C/G= 1, G/G = 1); *P*_rec_, *P*-value for recessive effect (C/C = 0, C/G = 0, G/G = 1).

**Table IV:** Association of rs2513280 (GAL) Genotypes With DXA-assessed Fat and Bone Mass (kg) in the ALSPAC Cohort

Fat/bone	Mean age (years)	N	C/C	C/G	G/G	*P*_add_	*P*_dom_	*P*_rec_
Fat	9.9	5,608	8.374 (4.729)	8.488 (5.086)	8.537 (5.029)	0.610	0.654	0.670
Bone			1.209 (0.188)	1.209 (0.205)	1.224 (0.199)	0.014	0.528	0.010
Fat	11.8	5,355	11.075 (6.008)	11.794 (6.857)	11.617 (6.662)	0.815	0.385	0.588
Bone			1.543 (0.277)	1.546 (0.302)	1.558 (0.291)	0.181	0.661	0.174
Fat	13.9	4,688	12.699 (7.497)	13.866 (8.011)	13.738 (8.002)	0.831	0.100	0.761
Bone			2.036 (0.367)	2.090 (0.412)	2.112 (0.404)	0.023	0.077	0.047
Fat	15.5	4,012	14.402 (8.663)	15.099 (9.062)	15.413 (9.135)	0.188	0.271	0.263
Bone			2.462 (0.436)	2.491 (0.476)	2.515 (0.467)	0.087	0.343	0.105

Genotype data are presented as mean (standard deviation).

*P*_add_, *P*-value for addictive effect (C/C = 0, C/G = 1, G/G = 2); *P*_dom_, *P*-value for dominant effect (C/C = 0, C/G= 1, G/G = 1); *P*_rec_, *P*-value for recessive effect (C/C = 0, C/G = 0, G/G = 1).

### Cognition

We analysed data collected from the WORD, IQ, Nonword repetition, WOLD and the Counting Span Working Memory Task tests as continuous variables (WORD median age: 7 years and 5 months; Counting Span Working Memory Task median age: 10 years and 7 months; all other measures taken at median age: 8 years and 7 months) (Table5). We found some evidence of association between rs2513280 and the offspring's results for the WOLD test (*P* = 0.032), although it was not robust enough to survive the correction for multiple comparisons.

**Table V:** Association of rs2513280 (*GAL)* Genotypes With Measures of Cognition in the ALSPAC cohort

Test	Mean age (years)	N	C/C	C/G	G/G	*P*_add_	*P*_dom_	*P*_rec_
WORD	7.5	5,986	28.060 (9.489)	28.409 (8.847)	28.472 (9.393)	0.657	0.627	0.734
IQ	8.7	5,517	104.946 (18.336)	104.109 (15.961)	105.042 (16.508)	0.124	0.914	0.076
Nonword	8.7	5,532	7.422 (2.360)	7.236 (2.499)	7.270 (2.522)	0.813	0.381	0.981
WOLD	8.7	5,561	7.419 (2.079)	7.391 (1.894)	7.550 (1.981)	0.032	0.455	0.030
Count–Span score	10.6	5,271	1.221 (0.220)	1.179 (0.341)	1.194 (0.320)	0.434	0.292	0.216
Count–Global score	10.6	5,271	18.520 (7.624)	18.315 (6.897)	18.355 (7.566)	0.274	0.927	0.205

Genotype data are presented as mean (standard deviation).

WORD, Wechsler Objective Reading Dimensions (number of correct words read (maximum of 55 words)); IQ, IQ test; Nonword, Nonword Repetition test (0–12 correct answers, children were asked to repeat 12 “nonwords”); WOLD, Listening comprehension and oral expression (0-15 correct answers); Count, Counting span working memory task; Span score (maximum score of 5 in increments of 0.5) and Global score (maximum score of 42).

*P*_add_, *P*-value for addictive effect (C/C = 0, C/G = 1, G/G = 2); *P*_dom_, *P*-value for dominant effect (C/C = 0, C/G= 1, G/G = 1); *P*_rec_, *P*-value for recessive effect (C/C = 0, C/G = 0, G/G = 1).

### Depression

Depression scores (median age: 7 years and 5 months, IQR: 7 years and 5 months–7 years and 6 months) based on the CIS-R questionnaire data were analysed as continuous and categorical variables, although neither suggested that rs2513280 was associated with depression using this criteria (*P* = 0.338 and *P* = 0.824 respectively, N = 3,420). Furthermore, using the ICD-10 diagnosis criteria to diagnose cases for depression provided no evidence of an association (OR = 1.086, *P* = 0.529, N = 3,408). Similarly, psychosis like symptoms (PLIKS) (median age: 11 years and 9 months, IQR: 11 years and 7 months–11 years and 10 months) was also not associated with rs2513280 (*P* = 0.373).

### Addiction

Results for analyses between rs2513280 and all addiction related phenotypes are presented in Table6.

**Table VI:** Association of rs2513280 (GAL) Genotypes With Addiction Related Traits in the ALSPAC Cohort

Phenotype	Mean age (years)	N	Case (%)	Control (%)	C/C (%)	C/G (%)	G/G (%)	OR	*P*_add_
CAST score	17.8	406	32.3	67.6	2.2	25.6	72.2	2.070	0.007
Frequency of cannabis use	17.8	169	48.6	51.4	2.3	23.7	74.0	2.243	0.018
≥1 Day ‘Binge drinking‘	15.5	3538	8.8	91.2	2.2	26.6	71.2	1.338	0.022
Frequency of alcohol use	15.5	2272	9.1	90.9	2.2	25.0	72.8	1.286	0.124
Frequency of alcohol use	17.8	1530	49.8	50.2	2.2	25.7	72.1	1.226	0.046
Daily versus non-daily cigarette smokers	17.8	878	20.2	79.8	2.2	26.0	71.8	1.001	0.997
PGSI score	17.8	2353	8.4	91.6	2.2	25.6	72.2	1.175	0.299

Genotype data are presented as percentage.

CAST, Cannabis abuse screening test—CAST Score of 1 or more = 1, CAST Score of 0 = 0 (having still used cannabis within the last 12 months).

Frequency of cannabis use, 4+ times per week = 1, 2–3 times per week = 0.

≥1 day ‘Binge drinking‘– participants were asked whether they spent a great deal of their day drinking alcohol over the last 2 years (yes = 1, no =0).

Frequency of alcohol use—drinking 2–3 times per week = 1, monthly ≥0.

PGSI, Problem gambling severity index—Problematic gambling exhibited in the past = 1, non-problem or no gambling in the past year = 0.

*P*_add_, *P*-value for addictive effect (C/C = 0, C/G = 1, G/G = 2).

#### Cannabis

When quantifying addiction using frequency of cannabis used (median age: 17 years and 9 months, IQR: 17 years and 7 months–17 years and 11 months) we observed strong evidence of an association when comparing very frequent users (4+ times per week) with occasional users (2–3 times per week) (OR = 2.243, *P* = 0.018, N = 169). We also observed increased odds when comparing heavy users (i.e. a CAST score of one or more) with occasional users (a CAST score of 0 despite using cannabis at least once in the last year) (OR = 2.070, *P* = 0.007, N = 406). However, as our sample size for these analyses were relatively small we sought out replication to strengthen the evidence that the observed signal was robust. The association of rs2513280 with the frequency of cannabis usage was analysed in the Yale-UPenn cohort (mean age = 40.0; range = 16–79 years) and the YNTR sample (mean age = 17.96; range = 14–27.5 years). Obtaining a similar phenotype definition to the one used in the ALSPAC cohort was challenging as the observation periods were different. The frequency of cannabis use was therefore analysed in the Yale-UPenn cohort by comparing subjects who used cannabis 12 days or less of the month with those who used cannabis 13 days or more per month. In the YNTR cohort, light cannabis users (1–4 times in the last month) were compared with heavy users (more than 20 times per month). For both, this was as comparable as we could get to our initial phenotype definition in the ALSPAC cohort. However, no discernible trend across genotypes was observed in either cohort (Yale-UPenn: AAs—OR = 1.10, *P* = 0.368, N = 2244, EAs—OR = 1.07, *P* = 0.620, N = 1840; YNTR: OR = 0.284, *P* = 0.138, N = 74).

#### Alcohol

At the age 15 clinic (median age: 15 years and 5 months, IQR: 15 years and 3 months–15 years and 7 months), teenagers were asked whether they spent a great deal of their day drinking alcohol over the last two years (i.e., at least one day ‘binge drinking’). Analysing this trait provided evidence to suggest rs2513280 was associated with this addiction-related phenotype (OR = 1.338, *P* = 0.022, N = 3538). Frequency of alcohol intake was also analysed at two time points by calculating the odds of those who reportedly drank alcohol 2–3 times per week compared to those who drank monthly or less. There was little evidence of an association with rs2513280 at age 14 (OR = 1.286, *P* = 0.124, N = 2272). However, at the age 17 clinic (median age: 17 years and 9 months, IQR: 17 years and 7 months–17 years and 11 months) there was nominal evidence of association (OR = 1.226, *P* = 0.046, N = 1530) although it was not robust enough to survive the correction for multiple comparisons. The relationships between rs2513280 and phenotypes based on alcohol intake were further examined to verify whether there was evidence of an interaction between genotype and gender. However, results from these analyses did little to support this in both the observed association with drinking for a great deal of the day (*P* = 0.288) and drinking alcohol 2–3 times per week compared to monthly or less (*P* = 0.314).

#### Smoking

Our analysis for both measurements of cotinine levels as continuous traits (median age: 17 years and 9 months, IQR: 17 years and 7 months–17 years and 11 months) resulted in little evidence of association with rs2513280 (Table7). Furthermore, dichotomising this phenotype using a cut-off of 50 ng/ml in order to calculate odds of daily smokers versus non-daily suggested rs2513280 did not have a strong effect on addiction using this trait (OR = 1.072, *P* = 0.620). There was no discernible trend across genotypes for frequency of cigarette smoking on a daily basis (*P* = 0.359, N = 373) and comparing the odds for those who smoked on a daily basis compared to non-daily smokers (OR = 1.00, *P* = 0.997, N = 878) suggest rs2513280 was not contributing to frequency of cigarette smoking. These results were concordant with large genome-wide association studies which found no evidence of association with cigarette smoking with SNPs in the *GAL* gene region (Tobacco and Genetics, 2010; David et al., 2012; Yoon et al., 2012).

**Table VII:** Association of rs2513280 (GAL) Genotypes With Cotinine Levels (ng/ml) Addiction Related Traits in the ALSPAC Cohort

Measurement	N	C/C	C/G	G/G	*P*_add_	*P*_dom_	*P*_rec_
1	1,428	42.428 (74.903)	33.627 (80.544)	38.765 (85.076)	0.457	0.763	0.361
2	1,428	43.281 (79.543)	34.402 (83.448)	41.540 (89.560)	0.283	0.831	0.210

Genotype data are presented as mean (standard deviation).

*P*_add_, *P*-value for addictive effect (C/C = 0, C/G = 1, G/G = 2); *P*_dom_, *P*-value for dominant effect (C/C = 0, C/G= 1, G/G = 1); *P*_rec_, *P*-value for recessive effect (C/C = 0, C/G = 0, G/G = 1).

#### Gambling

Comparing individuals who had shown signs of problematic gambling in the last year (i.e. a PGSI score of 1 or more) with those who did not, provided little evidence to suggest that rs2513280 was associated with gambling addiction (OR = 1.175, *P* = 0.299, N = 2353).

## DISCUSSION

We have undertaken extensive analyses to investigate the effects of a galanin enhancer genotype (rs2513280) on a range of phenotypes that are related to known functions of the galaninergic system (see recent reviews (Lang et al., 2007; Ogren et al., 2010; Picciotto et al., 2010)). Despite finding evidence of association with cannabis use in the ALSPAC cohort, we were unable to replicate this effect in the YNTR and Yale-UPenn cohorts. We found little evidence to suggest that SNP rs2513280 has an impact on any of the other traits analysed in our evaluations.

Galanin has been shown to increase alcohol consumption under a number of pre-clinical experimental paradigms. Administration of the neuropeptide into the paraventricular nucleus (PVN) or third ventricle potently increases alcohol intake in normal adult rats and those selected for high intake (Lewis et al., 2004; Rada et al., 2004; Schneider et al., 2007). Conversely, the non-selective galanin antagonist M40 decreases alcohol consumption and inhibits galanin-stimulated alcohol intake (Lewis et al., 2004; Rada et al., 2004). These findings may initially appear to be at odds with other studies showing that galanin decreases the responses to other drugs of abuse such as morphine, cocaine, and amphetamines (reviewed in (Picciotto et al., 2010)). It should be noted however that alcohol has a significant caloric value and may therefore utilise the same hypothalamic circuits that are known to mediate the stimulatory effects of galanin on food intake (Leibowitz et al., 2003; Schneider et al., 2007). Galanin injection into the PVN increases dopamine release in the nucleus accumbens (Rada et al., 1998) which would be consistent with the rewarding effects of alcohol (reviewed in (Picciotto et al., 2010)). In support of these rodent findings a number of studies have shown an association between alcoholism and SNPs in the genes for galanin or Gal_3_ receptor, but not Gal_1_ and Gal_2_ (Belfer et al., 2006; Belfer et al., 2007; Nikolova et al., 2013) and most recently, the GG haplotype has been shown to be associated with problem drinking in women (Nikolova et al., 2013). Of note, recent genome-wide association studies of alcohol use failed to identify any genetic variants in the galaninergic system that reached genome-wide significance (Kapoor et al., 2013; Gelernter et al., 2014). To date, we have been unable to identify any pre-clinical or clinical published studies that link galanin to cannabis usage.

The GG haplotype of the GAL5.1 enhancer region of the galanin gene has been evolutionary conserved and represents the major haplotype in human populations. Davidson et al. showed a 40% higher level of galanin expression than the CA allele in transfected cultured rat hypothalamic neurons (Davidson et al., 2011). The failure to replicate the initial association in the ALSPAC cohort of rs2513280 with the frequency of cannabis use in the Yale-UPenn and YNTR cohorts may be due in part to the difficulties we faced in phenotype harmonisation between the cohorts. Supplementary Tables SI and SIII compare the ALSPAC cohort with both replication samples and reflect the challenge we encountered for replication due to the disparity between samples, particularly for age and sample size. We attempted to dichotomise cannabis usage in the YNTR cohort to resemble the phenotype definition in the ALSPAC cohort as closely as possible; however this caused our replication sample size to become relatively small. Of significance, whilst initiation of cannabis use and its withdrawal are both heritable, little is known about the underlying genetic aetiology. Recent meta-analyses and genome-wide association studies of cannabis use with >10,000 individuals failed to identify any genetic variants that reached genome-wide significance (Le et al., 2009; Verweij et al., 2013a; Verweij et al., 2013b).

It is also important to focus on the lack of association of rs2513280 with the many other phenotypes that might have been expected to demonstrate a link with galanin, based on the pre-clinical rodent findings. We did not identify an association of rs2513280 with smoking, which is consistent with previous studies that have only shown an association with an intronic SNP in the Gal_1_ gene (Gold et al., 2012). Similarly, the absence of a relationship with body mass index, waist circumference, fat mass, and blood pressure are all consistent with other negative association studies previously reported for the galaninergic system (Schauble et al., 2005; Sutton et al., 2006). A previous study (Plaisier et al., 2009) demonstrated an association in Dutch, Finnish, and Mexican familial combined hyperlipidaemia families with an allele residing 5.6 kb upstream of the transcriptional start site of the *GAL* gene. The lack of an association of rs2513280 with fasting triglyceride levels in the ALSPAC cohort may be explained by the fact that the vast majority of the cohorts have normal triglyceride levels and thus are most unlikely to have a combined hyperlipidaemia. Further, it is possible that the two galanin enhancer regions which are more than 35 kb apart may differentially regulate galanin expression in differing tissues or cell types. We also failed to identify an association of rs2513280 with the various tests of cognition, and are unaware of any previous reports testing linkage of polymorphisms in any of genes involved in the galaninergic system with measures of cognition.

In contrast to the above, the lack of an association of rs2513280 with depression was initially surprising since Davidson et al., [2011] had shown it to be in LD with another closely located SNP (rs2156464, R^2^ = 0.687) in the galanin promoter which has been associated with major depressive disorder in adults (Wray et al., 2010). The lack of an association with depression in the ALSPAC cohort may be explained in part by the small sample size and the mean age of the subjects (17.8 years) when the depression scores were measured. At that time point only 8.4% of the cohort had an ICD-10 diagnosis for depression (whether mild, moderate or severe), and thus it is not surprising that we failed to identify an association with rs2513280. Further, it is possible that environmentally-induced epigenetic modification of GAL5.1 might be partly responsible for reducing the significance of some of these association studies. For example, hypomethylation of enhancer regions that regulate neuropeptide expression in the hypothalamus have been shown to be strongly influenced by early life stress (Murgatroyd et al., 2009).

Other SNPs within the *GAL* gene region to have been previously implicated in disease susceptibility include rs2187331 (R^2^ = 0.687 with rs2513280), which has been linked with increased triglyceride levels (16), and rs948854 (R^2^ = 0.462 with rs2513280) which has shown evidence of association with opioid dependence (Beer et al., 2013). As we did not find evidence to suggest that rs2513280 was implicated in triglyceride synthesis and as it was not in high linkage disequilibrium with rs948854. Other previously reported SNPs within the *GAL* gene region did not appear to be in linkage disequilibrium with rs2513280 (rs694066 (R^2^ = 0.007) and rs7101947 (R^2^ = 0.135)) (Ruano et al., 2006).

According to HapMap there seems to be some variation between the minor allele frequencies of rs2513280 across different ethnic groups. As the ALSPAC cohort consists predominantly of individuals with a maternally reported European ethnic origin we were not able to robustly evaluate how this variation may affect the results of our study. However, it is important for future studies that investigate the effects of rs2513280 using a more ethnically diverse sample to adjust for this possible population stratification accordingly.

In summary, we have used a large longitudinal cohort of over 10,000 young adults and failed to demonstrate an association between a SNP in a galanin enhancer region, which predicts an increase in galanin expression, with any of the pathophysiological parameters measured. Our results also illustrate the importance of seeking independent replication of initial promising findings, even when these are informed by relevant neurobiology and preclinical data (Munafo and Gage, 2013). It is increasingly clear that the contribution of individual common genetic variants to complex behavioural traits will be very small, so that very large samples will be required to reliably detect these (Munafo and Flint, 2011). Whilst genome-wide methods are hampered by the need to harmonise phenotypes across contributing studies, and the requirement to achieve genome-wide significance, these have typically generated more reproducible findings than candidate gene methods (Flint and Munafo, 2013).

## Supporting Information

Table SI. Overview of Phenotypes Analyzed in the ALSPAC Cohort Table SII. Comparison of Inclusion Criteria, Case, and Control Definitions for All 3 Cohorts Table SIII. Comparison of Genotypes Frequencies Across Cohorts Table SIV. Comparison of Cases and Controls by Genotype Frequencies For All ‘Addiction’ Related Phenotypes
