# Supplementary material for: Evaluating the role of a galanin enhancer genotype on a range of metabolic, depressive and addictive phenotypes
Source: Am J Med Genet B Neuropsychiatr Genet. 2014 Sep 16;165(8):654–64. doi: 10.1002/ajmg.b.32270 (PMC4388908; doi:10.1002/ajmg.b.32270)
Supplement: Supplementary file 1 — Table SI. Overview of Phenotypes Analyzed in the ALSPAC Cohort Table SII. Comparison of Inclusion Criteria, Case, and Control Definitions for All 3 Cohorts Table SIII. Comparison of Genotypes Frequencies Across Cohorts Table SIV. Comparison of Cases and Controls by Genotype Frequencies For All ‘Addiction’ Related Phenotypes [file ajmg0165-0654-sd1.docx]

Supplementary Material

Supplementary Table 1: Overview of Phenotypes analysed in the ALSPAC Cohort

| **Mean Age (Years)** | **Category** | **Phenotype** | **Description** |
| --- | --- | --- | --- |
| 7.6 | Metabolism | BMI | Continuous – Body Mass Index – kg/m^2^ |
|  |  | Waist Circumference | Continuous – measured to the nearest 1 mm at the mid-point between the lower ribs and the pelvic bone |
|  |  | Systolic Blood Pressure | Continuous – taken at rest, average of 2 readings |
|  |  | Diastolic Blood Pressure | Continuous – taken at rest, average of 2 readings |
|  | Cognition | WORD | Continuous - Wechsler Objective Reading Dimensions (number of correct words read (maximum of 55 words)) |
|  | Depression | CIS-R assessed Depression Score (Continuous) | Continuous – obtained from computerised self-reported questionnaire for the revised version of the Clinical Interview Schedule (CIS-R). |
|  |  | CIS-R assessed Depression Score (Categorical) | Categorical – obtained from the above, dichotomised so that scores ≥ 12 = 1, scores < 12 = 0 |
|  |  | ICD-10 diagnosed Depression | Categorical – yes/no – derived from CIS-R data |
| 8.7 | Cognition | IQ Test | Continuous - measured using the Weschler Intelligence Scale for Children |
|  |  | Nonword | Continuous - Nonword Repetition test (0 – 12 correct answers, children were asked to repeat 12 “nonwords”) |
|  |  | WOLD | Continuous - Listening comprehension and oral expression (0-15 correct answers) |
| 9.9 | Metabolism | BMI | Continuous – Body Mass Index – kg/m^2^ |
|  |  | Waist Circumference | Continuous – measured to the nearest 1 mm at the mid-point between the lower ribs and the pelvic bone |
|  |  | Systolic Blood Pressure | Continuous – taken at rest, average of 2 readings |
|  |  | Diastolic Blood Pressure | Continuous – taken at rest, average of 2 readings |
|  |  | DXA-assessed Fat mass | Continuous - Whole body dual-emission X-ray absorptiometry (DXA) determined fat mass |
|  |  | DXA-assessed Bone mass | Continuous - Whole body dual-emission X-ray absorptiometry (DXA) determined bone mass |
| 10.7 | Metabolism | BMI | Continuous – Body Mass Index – kg/m^2^ |
|  |  | Waist Circumference | Continuous – measured to the nearest 1 mm at the mid-point between the lower ribs and the pelvic bone |
|  | Cognition | Counting Span Working Memory Task (Span Score) | Continuous - maximum score of 5 in increments of 0.5 |
|  |  | Counting Span Working Memory Task (Global Score) | Continuous - maximum score of 42 |
| 11.8 | Metabolism | BMI | Continuous – Body Mass Index – kg/m^2^ |
|  |  | Waist Circumference | Continuous – measured to the nearest 1 mm at the mid-point between the lower ribs and the pelvic bone |
|  |  | Systolic Blood Pressure | Continuous – taken at rest, average of 2 readings |
|  |  | Diastolic Blood Pressure | Continuous – taken at rest, average of 2 readings |
|  |  | DXA-assessed Fat mass | Continuous - Whole body dual-emission X-ray absorptiometry (DXA) determined fat mass |
|  |  | DXA-assessed Bone mass | Continuous - Whole body dual-emission X-ray absorptiometry (DXA) determined bone mass |
|  | Depression | Psychosis like symptoms (PLIKS) combined score | Continuous - semi-structured interview consisting of 12 core questions |
| 13.9 | Metabolism | BMI | Continuous – Body Mass Index – kg/m^2^ |
|  |  | Waist Circumference | Continuous – measured to the nearest 1 mm at the mid-point between the lower ribs and the pelvic bone |
|  |  | DXA-assessed Fat mass | Continuous - Whole body dual-emission X-ray absorptiometry (DXA) determined fat mass |
|  |  | DXA-assessed Bone mass | Continuous - Whole body dual-emission X-ray absorptiometry (DXA) determined bone mass |
| 15.5 | Metabolism | BMI | Continuous – Body Mass Index – kg/m^2^ |
|  |  | Waist Circumference | Continuous – measured to the nearest 1 mm at the mid-point between the lower ribs and the pelvic bone |
|  |  | Systolic Blood Pressure | Continuous – taken at rest, average of 2 readings |
|  |  | Diastolic Blood Pressure | Continuous – taken at rest, average of 2 readings |
|  |  | DXA-assessed Fat mass | Continuous - Whole body dual-emission X-ray absorptiometry (DXA) determined fat mass |
|  |  | DXA-assessed Bone mass | Continuous - Whole body dual-emission X-ray absorptiometry (DXA) determined bone mass |
|  | Addiction | ≥ 1 Day ‘Binge Drinking | participants were asked whether they spent a great deal of their day drinking alcohol over the last 2 years (yes = 1, no =0) |
|  |  | Frequency of Alcohol Use | drinking 2-3 times per week = 1, monthly or less =0 |
| 17.8 | Addiction | Cotinines - continuous | Continuous |
|  |  | Cotinines – dichotomised | Categorical – a cut-off of 50 ng/ml in order to calculate odds of daily smokers vs non-daily |
|  |  | CAST Score | Cannabis abuse screening test – CAST Score of 1 or more = 1, CAST Score of 0 = 0 (having still used cannabis within the last 12 months) |
|  |  | Frequency of Cannabis Usage | 4+ times per week = 1, 2-3 times per week = 0 |
|  |  | Frequency of Alcohol Use | drinking 2-3 times per week = 1, monthly or less =0 |
|  |  | Daily vs Non-Daily Cigarette Smokers | Categorical – yes/no – based on questionnaire data |
|  |  | PGSI score | Problem gambling severity index – Problematic gambling exhibited in the past = 1, non-problem or no gambling in the past year = 0 |

Supplementary Table 2: Comparison of Inclusion criteria, case and control definitions for all 3 cohorts

| **Cohort** | **Inclusion Criteria** | **Case Definition** | **Control Definition** |
| --- | --- | --- | --- |
| ALSPAC | Pregnant women, their partners and their children born in 1991-1992. Offspring who attended the Teen Focus 4 clinic (Mean Age 17.8 years) participants were asked to complete a six item cannabis abuse screen test (CAST) ([Legleye and others 2012](#_ENREF_20)) which entailed questions about cannabis use in the past year. This outcome was dichotomised for our analysis to distinguish between heavy cannabis users (i.e. a CAST score of 1 or more) and infrequent cannabis users. Subjects who were successfully for SNP rs2513280 and had used cannabis at least once in the last 12 months were included for analysis. | Subjects with a CAST score ≥ 1 (heavy cannabis users) | Subjects with a CAST score = 0 (infrequent cannabis users) |
| Yale-UPenn (AAs) | Families were ascertained from treatment centers and advertisements that recruited affected sibling pairs (ASPs) meeting Diagnostic and Statistical Manual of Mental Disorders, 4th Edition (DSM-IV) criteria for cocaine dependence or opioid dependence. Probands were excluded from further study if they had ever received a clinical diagnosis of a major psychotic illness (for example, schizophrenia or schizoaffective disorder). Other family members of the ASPs were recruited when available, regardless of affection status. The case–control subjects were recruited from substance abuse treatment centers and through advertisements at the University of Connecticut Health Center, Yale University School of Medicine, the Medical University of South Carolina, the University of Pennsylvania, and McLean Hospital. | Subjects who used cannabis 13 days or more per month | Subjects who used cannabis 12 days or less of the month |
| YNTR | Since 1987, newborn twins enter the register and surveys were collected that are conditional on the age of the children. In the past 25 years, we have collected data from 70,784 children (i.e., for all children there is at least one survey available).  At age 12, data from the twins themselves was collected, including measures on frequency of cannabis use, collected at age 14, 16 and 18. Individuals analysed in the replication were successfully genotyped for SNP rs2513280 and a measure of cannabis usage in the last month. | Heavy cannabis users ( categorized as individuals who used cannabis more than 20 times per month) | Light cannabis users (1-4 times in the last month) were used as the baseline |

Supplementary Table 3: Comparison of genotypes frequencies across cohorts

| **Cohort** | **Mean Age (Range)** | **N** | **% Male** | **MAF** | **C/C** | **C/G** | **G/G** | **OR (SE)** | **P** |
| --- | --- | --- | --- | --- | --- | --- | --- | --- | --- |
| ALSPAC | 17.8 (16.3 – 20.0) | 406 | 50.1% | 0.15 | 2.2% | 25.6% | 72.2% | 2.070 (0.559) | 0.007 |
| Yale-UPenn (AAs) | 40.0 (16.0 – 79.0) | 2244 | 56.7% | 0.18 | 3.3% | 29.5% | 67.2% | 1.1 (0.105) | 0.368 |
| Yale-UPenn (EAs) | 40.0 (16.0 – 79.0) | 1840 | 60.5% | 0.15 | 2.2% | 25.5% | 72.3% | 1.07 (0.143) | 0.620 |
| YNTR | 17.96 (14 – 27.5) | 74 | 66.2% | 0.13 | 1.3% | 23.0% | 75.7% | 0.284 (0.848) | 0.138 |

MAF – Minor Allele Frequency

OR – Odds Ratio

SE – Standard Error

Supplementary Table 4: Comparison of cases and controls by genotype frequencies for all ‘addiction’ related phenotypes

| **Phenotype** | **Mean Age (years)** | **Case/Control** | **N** | **C/C** | **C/G** | **G/G** | **OR** | **P_add_** |
| --- | --- | --- | --- | --- | --- | --- | --- | --- |
| CAST score | 17.8 | Case  Control | 131  275 | 1.5%  2.5% | 18.3%  34.2% | 80.2%  63.3% | 2.070 | 0.007 |
| Frequency of Cannabis Use | 17.8 | Case  Control | 87  82 | 2.4%  2.3% | 12.2%  32.2% | 85.4%  65.5% | 2.243 | 0.018 |
| ≥ 1 Day ‘Binge Drinking‘ | 15.5 | Case  Control | 312  3226 | 1.3%  2.3% | 22.1%  27.0% | 76.6%  70.7% | 1.338 | 0.022 |
| Frequency of Alcohol Use | 15.5 | Case  Control | 190  2082 | 1.6%  2.2% | 21.0%  25.7% | 77.4%  72.1% | 1.286 | 0.124 |
| Frequency of Alcohol Use | 17.8 | Case  Control | 762  768 | 1.8%  2.5% | 23.5%  27.3% | 74.7%  70.2% | 1.226 | 0.046 |
| Daily vs Non-Daily Cigarette Smokers | 17.8 | Case  Control | 177  691 | 2.8%  1.4% | 23.7%  28.7% | 73.4%  69.9% | 1.001 | 0.997 |
| PGSI score | 17.8 | Case  Control | 197  2156 | 1.5%  2.2% | 23.4%  25.8% | 75.1%  72.0% | 1.175 | 0.299 |

Genotype data are presented as percentage

CAST – Cannabis abuse screening test – CAST Score of 1 or more = 1, CAST Score of 0 = 0 (having still used cannabis within the last 12 months)

Frequency of Cannabis Use – 4+ times per week = 1, 2-3 times per week = 0

≥ 1 Day ‘Binge Drinking‘- participants were asked whether they spent a great deal of their day drinking alcohol over the last 2 years (yes = 1, no =0)

Frequency of Alcohol Use – drinking 2-3 times per week = 1, monthly or less =0

PGSI - Problem gambling severity index – Problematic gambling exhibited in the past = 1, non-problem or no gambling in the past year = 0

P_add_ – P-value for additive effect (C/C = 0, C/G = 1, G/G = 2)
